# *Puerariae lobatae* radix protects against UVB-induced skin aging *via* antagonism of REV-ERBα in mice

**DOI:** 10.3389/fphar.2022.1088294

**Published:** 2022-12-22

**Authors:** Luyao Ma, Meiping Huang, Guanghui Sun, Yanke Lin, Danyi Lu, Baojian Wu

**Affiliations:** ^1^ Institute of Molecular Rhythm and Metabolism, Guangzhou University of Chinese Medicine, Guangzhou, China; ^2^ College of Pharmacy, Jinan University, Guangzhou, China

**Keywords:** Puerariae lobatae radix, skin aging, REV-ERBα, BMAL1, Nrf2

## Abstract

*Puerariae lobatae* radix (PLR) is a wildly used herbal medicine. Here we aimed to assess the PLR efficacy against UVB (ultraviolet-B)-induced skin aging and to determine the mechanisms thereof. We found a significant protective effect of PLR (topical application) on UVB-induced skin aging in mice, as evidenced by reduced skin wrinkles, epidermal thickness, and MDA (malondialdehyde) content as well as increased levels of HYP (hydroxyproline) and SOD (superoxide dismutase) in the skin. In the meantime, *Mmp-1, p21* and *p53* levels were decreased in the skin of PLR-treated mice. Anti-aging effects of PLR were also confirmed in L929 cells. Furthermore, PLR up-regulated skin expression of BMAL1*,* which is a known regulator of aging by promoting *Nrf2* and antioxidant enzymes. Consistently, *Nrf2* and several genes (i.e., *Prdx6*, *Sod1*, and *Sod2*) encoding antioxidant enzymes in the skin were increased in PLR-treated mice. Moreover, based on Gal4 chimeric assay, *Bmal1* reporter gene and expression assays, we identified PLR as an antagonist of REV-ERBα that can increase *Bmal1* expression. Intriguingly, loss of *Rev-erbα* protected mice against UVB-induced skin aging and abrogated the protective effect of PLR. In conclusion, PLR acts as an antagonist of REV-ERBα and promotes the expression of BMAL1 to protect against skin aging in mice.

## Introduction

Aging is an inevitable process for all living organisms. As the skin is directly exposed to the environment, skin aging is relatively more rapid due to environmental insults such as chemicals and ultraviolet radiation (Tobin, 2017). Skin aging is characterized by an increase in the epidermal thickness, collagen degradation and loss of subcutaneous fat (Kohl et al., 2011; Tian et al., 2011; Khare et al., 2021). Although skin aging is regarded as a cosmetic problem, it can result in disfigurement and skin diseases (such as wound healing and tumor progression) and has profound psychological consequences (Worley, 2006; Bissell and Hines, 2011; Shao et al., 2017). To date, there are two major classes of agents for management of skin aging, namely, antioxidants and cell regulators (Zouboulis et al., 2019). However, these medications (e.g., retinoid and coenzyme Q10) are concerned with poor efficacy and/or adverse effects (Kwon et al., 2019). Therefore, it is of interest to search for more effective and safer therapeutic agents.

Although the etiologies of premature skin aging (or photoaging) are not fully elucidated, several contributing factors have been identified, including oxidative stress [i.e., formation of reactive oxygen species (ROS)], mutations of mitochondrial DNA, and shortening of telomeres (Beani, 2014; Papaccio et al., 2022; Son et al., 2022). Notably, ROS exerts a critical role in photoaging. ROS leads to increased skin expression of matrix metalloproteinases (MMPs, which degrade collagen within the extracellular matrix) through activation of the transcription factor AP-1 (activation protein 1) (Kim et al., 2018). On the other hand, AP-1 activation inhibits the activity of transforming growth factor β (TGF-β), a major regulator of collagen synthesis in the skin, and thus suppresses neocollagenesis (Oh et al., 2021). In addition, deletion of mitochondrial DNA can cause oxidative stress, which in turn induces mitochondrial mutations. This interaction appears to exacerbate the detrimental influences of ROS on the skin (Suski et al., 2018; Wikramanayake et al., 2022).

BMAL1 (brain and muscle ARNT-like protein 1) and REV-ERBα (also known as NR1D1, nuclear receptor subfamily 1 group D member 1) are two transcription factors that play pivotal roles in generation of circadian rhythms (i.e., ∼24 h oscillations in physiology and behaviors) in mammals (Mohawk et al., 2012). BMAL1 functions as a key positive element of the molecular clock and activates the transcription of several other clock genes (such as *Pers* and *Crys*) and clock-controlled genes by forming a heterodimer with CLOCK (circadian locomotor output cycles kaput) protein. In contrast, REV-ERBα regulates circadian rhythms through direct inhibition of BMAL1 expression (as a transcriptional target of REV-ERBα). Mechanistically, REV-ERBα binds to a RevRE responsive element located in gene promoters and subsequently recruits two corepressors (nuclear corepressor 1 and histone deacetylase 3), and ultimately leads to transcription suppression of target genes including *Bmal1* (Takahashi, 2017; Patke et al., 2020).

In addition to regulating circadian rhythms, BMAL1 and REV-ERBα have been implicated in regulation of many types of diseases such as metabolic syndrome, obesity, inflammatory diseases (e.g., fulminant hepatitis, colitis, lung inflammation and acne), neurodegenerative diseases, and cancers (Wang et al., 2018; Keshvari et al., 2020; Pan et al., 2020; Yu et al., 2021; Zhang et al., 2021). Notably, BMAL1 and REV-ERBα are also involved in the aging process. *Bmal1* ^−/−^ mice show premature aging phenotypes such as reduced lifespan, cataracts, reduced subcutaneous fat, and organ shrinkage (Kondratov et al., 2006). Further studies revealed that *Bmal1* regulation of aging is attained mainly through modulation of oxidative stress and ROS homeostasis (Zhang et al., 2022). *Bmal1* promotes oxidative defense by directly and indirectly up-regulating the expression of major antioxidant enzymes such as SOD (superoxide dismutase), PRDXs (peroxiredoxines) and GPX (glutathione peroxidase) (Töbelmann and Dittmar, 2021). Indirect pathway involves activation of NRF2 (a master regulator of anti-oxidative responses) by BMAL1 (Chhunchha et al., 2020). Consistent with a protective role of BMAL1 in aging, REV-ERBα (a negative regulator of BMAL1) has been shown to promote the aging of bone mesenchymal stem cells and oncogene-induced senescence (He et al., 2015; Sulli et al., 2018). Therefore, BMAL1 and REV-ERBα are regarded as promising drug targets for premature aging. Notably, REV-ERBα is more advantageous in terms of druggability because it has a ligand-binding pocket and thus its activity can be readily modified by small molecules (Burris, 2008).

*Puerariae lobatae* radix (PLR, also known as *Gegen* in Chinese) is the dried root of *Pueraria lobate* (Willd.) Ohwi, which is traditionally used in east Asia to treat various disorders such as fever, diarrhea, muscle stiffness, hypertension, and diabetes (Wong et al., 2011; Zhang et al., 2013; Chinese Pharmacopoeia Commission, 2020). Isoflavones such as puerarin, daidzein and daidzin are a major class of active ingredients in PLR, and demonstrate a variety of health-promoting effects, including antihypertension, antidiabetic, anti-alcoholism, and neuroprotective properties (Zhang et al., 2013). For instance, puerarin suppresses blood pressure by activating endothelial nitric oxide synthase (Shi et al., 2019). Puerarin treatment leads to significant increases in expression levels of peroxisome proliferator-activated receptor γ and its downstream targets such as adiponectin and glucose transporter 4, resulting in enhanced cellular glucose utilization (Lee et al., 2010). Daidzein and daidzin suppress ethanol intake probably by inhibiting acetaldehyde dehydrogenase 2, which is essential for the oxidation of acetaldehyde derived from ethanol metabolism (Zhang et al., 2017). However, it remains largely unknown whether PLR can be used to manage skin problems such as photoaging.

Here, we aimed to assess the PLR efficacy against UVB-induced skin aging and to determine the mechanisms. Anti-photoaging effects of PLR were examined in both UVB-treated mice and cells. The extent of aging was assessed by analyzing HYP (hydroxyproline), SOD (superoxide dismutase), MDA (malondialdehyde), ROS and *Mmp-1/p21/p53* in the skin. The antagonistic ability of PLR against REV-ERBα was measured using Gal4 chimeric assay, *Bmal1* reporter gene and expression assays. We demonstrated that PLR acts as an antagonist of REV-ERBα and promotes the expression of BMAL1 to protect against skin aging in mice.

## Materials and methods

### Materials

PLR, puerarin and daidzein were purchased from Biopurify Phytochemicals (Chengdu, China). PLR was validated by Prof. Baojian Wu at Guangzhou University of Chinese Medicine. Vitamin C (VC) was obtained from Yuanye Biotechnology (Shanghai, China). SR8278 was obtained from MedChemExpress (Monmouth Junction, NJ). Biochemical kits for SOD, MDA and HYP were purchased from Jiancheng Bioengineering Institute (Nanjing, China). Biochemical kit for ROS was obtained from Beyotime Biotechnology (Shanghai, China).

### Preparation of PLR extract

200 g of PLR was soaked in 1,000 ml of 80% ethanol solution for 30 min. After reflux and suction filtration, the filtrate was concentrated to 100 ml in vacuum, and then freeze-dried to powder. The extraction yield was 10.5%. The contents of puerarin and daidzein in PLR extract powder were estimated to be 20.8% and 3.16%, respectively, according to a classic LC-MS/MS method (Supplementary Table S1; Supplementary Figure S1 in supplementary material). For cell experiments, PLR extract powder was made into a stock solution (50 mg/ml in DMSO). For animal experiments, PLR extract powder was made into an emulsion (20 mg/ml) as previously described (Ali et al., 2019).

### Animal studies

C57BL/6 mice (male, eight-week-old) were purchased from Guangzhou Carrot Biotechnology (Guangzhou, China). *Rev-erbα* ^−/−^ mice (C57BL/6 background) have been described (Zhang et al., 2019). All mice were maintained on a standard 12 h/12 h light/dark cycle (light on from 07:00 a.m. to 19:00 p.m. and light off from 19:00 p.m. to 07:00 a.m.) with *ad libitum* intake of food and water. All animal experiments were conducted with the approval of the Animal Ethics Committee of Guangzhou University of Chinese Medicine (License number: 20210225033, approved on 01/03/2021).

Mice with shaved back were grouped randomly as follows: (1) control group (*n* = 6); (2) UVB group (*n* = 6): mouse back skin was irradiated with UVB (120 mJ/cm^2^) daily and blank emulsion was used to smear the back after irradiating; (3) VC group (*n* = 6): the emulsion containing VC (40 mg/kg) was used to smear the back after irradiating; and (4) PLR groups, which were divided into three subgroups: low dose group (*n* = 6, 25 mg/kg), middle dose group (*n* = 6, 100 mg/kg) and high dose group (*n* = 6, 400 mg/kg). Mice in PLR groups were smeared with the emulsion containing PLR extract after irradiating. The source of radiation was a narrow band UVB bulb (Philips model PL-9 9W/01/2P) emitting photons with wavelengths between 306 and 316 nm. The radiation flux was measured by using an UV irradiance meter (LS125, Linshang Technology, Shenzhen, China). All mice were treated for 45 days and sacrificed at 14:00 p.m. to collect skin samples for further assays.

### Cell culture and treatment

L929 mouse fibroblast cells (Procell Biotechnology, Wuhan, China) were used to assess the protective effects of PLR and VC on UVB-induced cell senescence. Cells were cultured in MEM (Procell Biotechnology, PM150410) supplemented with 100 μg/ml streptomycin, 100 U/mL penicillin and 10% FBS (Procell Biotechnology, 164210-50). Once reaching a confluence of ∼90%, cells were subjected to UVB irradiation (100 mJ/cm^2^) and then treated with VC (6 μg/ml) or PLR extract (0.1, 0.5 or 1 μg/ml). 24 h later, L929 cells were collected for further assays.

### Hematoxylin & eosin (H&E) staining

Briefly, skin samples were fixed, dehydrated, embedded, and cut into four-μm-thick slices. For histopathological evaluations, skin slices were stained with hematoxylin and eosin and photographed with a Leica DM750 microscope (Leica Microsystems, Switzerland).

### SA-β-gal assay

A SA-β-gal staining kit (Beyotime, Shanghai, China) was used to detect the β-galactosidase activity. Following VC or PLR treatment, L929 cells were fixed with β-galactosidase fixation solution. After washing twice with PBS, cells were incubated with 1 ml staining solution at 37°C overnight. Staining images were pictured by a Leica DM750 microscope (Leica Microsystems, Switzerland).

### Measurements of HYP, SOD, and MDA

Skin samples were homogenized in lysis buffer using a homogenizer (Jingxin Technologies, Shanghai, China). Tissue lysate was centrifuged (10000 ×*g*) for 10 min. The supernatant was collected to determine the contents of HYP, SOD and MDA using their respective assay kits.

### Cell viability assay

The cell viability was detected by CCK-8 assay (Beyotime, Shanghai, China). Following VC or PLR treatment, L929 cells were cultured with 10 µL CCK-8 solution and 100 µL fresh medium for 30 min. The absorbance at 450 nm was monitored by a BioTek Synergy H1 Multi-Mode Microplate Reader (BioTek Instruments, Bad Friedrichshall, Germany).

### Measurement of intracellular ROS

DCFH-DA was used as a probe to measure intracellular ROS. In brief, following VC or PLR treatment, L929 cells were loaded with DCFH-DA (10 μM in serum-free medium) and incubated at 37°C for 20 min. Real-time fluorescence was monitored using a BioTek Microplate Reader at 488 nm excitation and 525 nm emission wavelengths.

### Gal4 co-transfection assay

Gal4 co-transfection assay was performed as described in our previous publication (Wang et al., 2019). Briefly, pGL4.35-Luc reporter (100 ng), Gal4-REV-ERBα-LBD plasmid (200 ng) and pRL-TK vector (10 ng) were co-transfected into L929 cells using Lipofectamine 3,000 (Thermo Fisher Scientific, Waltham, United States). 18 h after transfection, cells were treated with SR8278 (10 µM) or PLR (1 μg/ml) or vehicle for another 24 h. A reporter assay system (Promega, Walldorf, Germany) was used to detect the cellular luciferase activity.

### Luciferase reporter assay

pRL-TK vector (10 ng) and *Bmal1*-Luc reporter plasmid (250 ng) were co-transfected into L929 cells. The luciferase activities were measured as described in the “Gal4 co-transfection assay” section.

### qPCR assay

Total RNAs in skin and cell samples were extracted using RNAex Pro Reagent (Accurate Biology, Guangzhou, China). Reverse transcription was performed using the Evo M-MLV RT kit (Accurate Biology, Guangzhou, China). qPCR reaction, with a total reaction volume of 20 μL and 5 ng cDNA, was performed using the 2X SYBR Green Pro Taq HS Premix (Accurate Biology, Guangzhou, China). The conditions of quantitative PCR were as follows: 40 cycles at 95°C for 5 s, 60°C for 15 s and 72°C for 10 s. *Gapdh* was used as an internal standard. The primers used are listed in Table 1.

**TABLE 1:** Primer sequences used for qPCR assays.

Gene	Forward (5′–3′)	Reverse (5′–3′)
*Mmp-1*	CTT​CTT​CTT​GTT​GAG​CTG​GAC​TC	CTG​TGG​AGG​TCA​CTG​TAG​ACT
*p21*	TTA​GAA​CGC​TTA​AAT​GCC​GGA​G	GAT​GAA​GCT​GAC​GAA​CTG​CCT
*p53*	GTC​ACA​GCA​CAT​GAC​GGA​GG	TCT​TCC​AGA​TGC​TCG​GGA​TAC
*Rev-erbɑ*	TTT​TTC​GCC​GGA​GCA​TCC​AA	ATC​TCG​GCA​AGC​ATC​CGT​TG
*Bmal1*	CTC​CAG​GAG​GCA​AGA​AGA​TTC	ATA​GTC​CAG​TGG​AAG​GAA​TG
*Cry1*	CAC​TGG​TTC​CGA​AAG​GGA​CTC	CTG​AAG​CAA​AAA​TCG​CCA​CCT
*Cry2*	CAC​TGG​TTC​CGC​AAA​GGA​CTA	CCA​CGG​GTC​GAG​GAT​GTA​GA
*Per1*	GAT​GTG​GGT​GTC​TTC​TAT​GGC	AGG​ACC​TCC​TCT​GAT​TCG​GC
*Per2*	TTG​ACG​CGG​CGA​AGC​GGT​GAG​TG	GGG​ACG​CAG​TGT​GAA​CCT​GG
*Nrf2*	TCT​CCT​CGC​TGG​AAA​AAG​AA	AAT​GTG​CTG​GCT​GTG​CTT​TA
*Prdx6*	TTC​AAT​AGA​CAG​TGT​TGA​GGA​TCA	CGT​GGG​TGT​TTC​ACC​ATT​G
*Sod1*	CAG​GAC​CTC​ATT​TTA​ATC​CTC​AC	TGCCCAGGTCTCCAACAT
*Sod2*	TGC​TCT​AAT​CAG​GAC​CCA​TTG	GTA​GTA​AGC​GTG​CTC​CCA​CAC
*Gapdh*	CAA​GGA​GTA​AGA​AAC​CCT​GGA	CGA​GTT​GGG​ATA​GGG​CCT​CT

### Western blotting

Lysed proteins from skin tissues and cells were subjected to SDS-polyacrylamide gel electrophoresis and then transferred onto a polyvinylidene difluoride membranes (Millipore, Bedford, MA). The membranes were incubated with primary antibodies (anti-BMAL1, 1:2000, proteintech, 14268-1-AP) overnight at 4°C, then with secondary antibodies (1:10,000, Lablead, RN0100) for 1 h at room temperature. Immunoreactive bands were visualized by using an enhanced luminescence kit (Merck-Millipore, Bedford, MA) and a Tanon Imaging System (Tanon, Shanghai, China), and quantified with ImageJ software (NIH, Bethesda, MD). GAPDH was used as an internal standard.

### Statistical analysis

Data are expressed as the means ± standard deviation. Statistical differences between the means of two groups were analyzed by Student’s t-test. *p* < 0.05 (*) was considered to be significantly different.

## Results

### A protective effect of PLR on UVB-induced skin aging in mice

**FIGURE 1:**
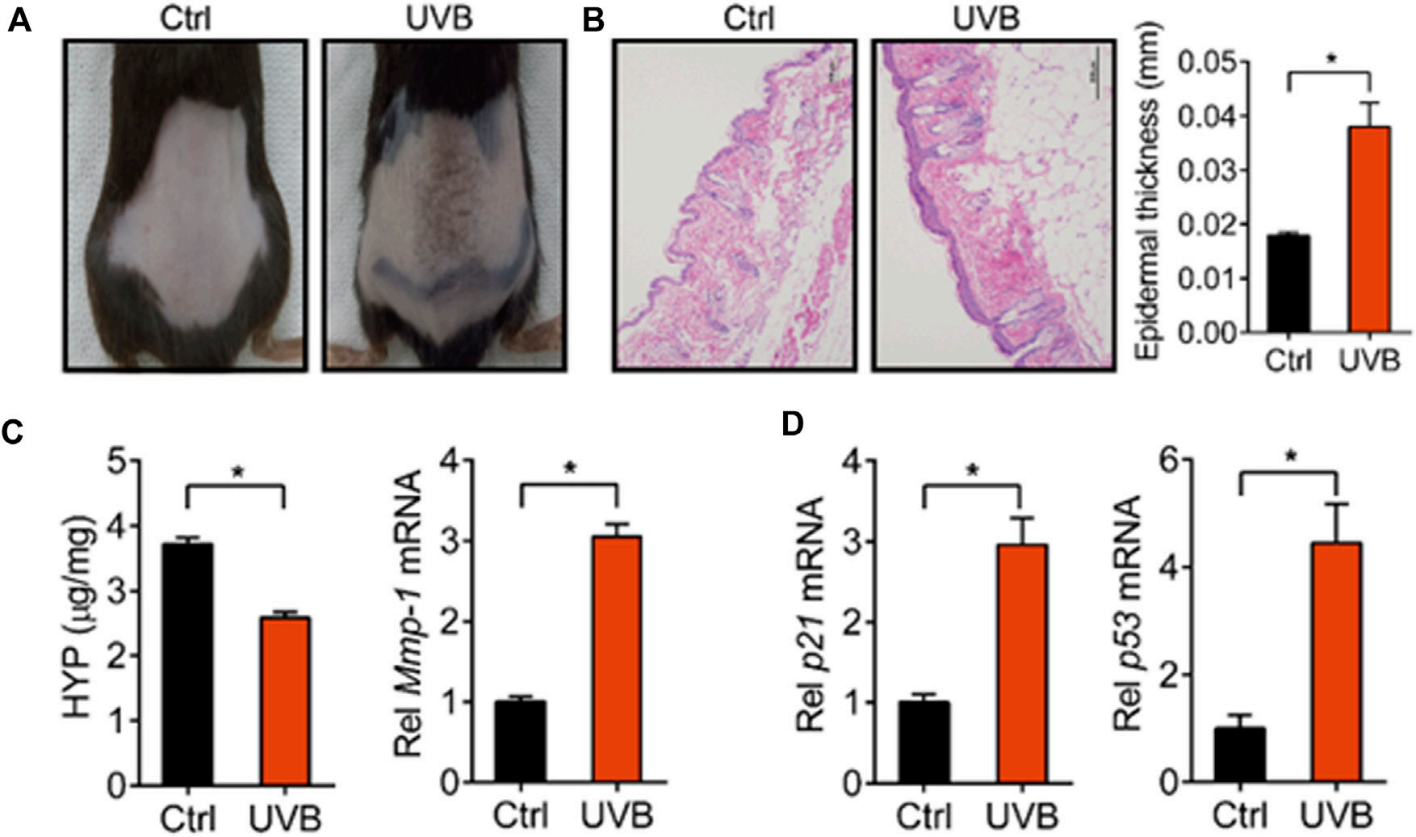
Characterization of UVB-induced skin aging model. **(A)** Skin photographs taken after irradiating the back of mice with UVB for 45 d. **(B)** Representative images of H&E staining and quantitative analysis of the epidermal thickness. Scale bar: 200 µm. **(C)** Content of HYP and expression level of *Mmp-1* mRNA in the skin. **(D)** Expression levels of *p21* and *p53* mRNA in the skin. Data are presented as mean ± SD (*n* = 6). *p < 0.05. Ctrl, control.

We first established a skin aging model by irradiating the back of mice with UVB. After 45 days, we found that the mice irradiated with UVB showed aging signs on the back skin, such as wrinkling, roughness, melanosis, and epidermal thickening (Figures 1A,B). HYP is an important component of fibrillar collagen and MMP-1 degrades collagen fibers in extracellular matrix, both of which are closely related to skin aging (Li and Wu, 2018; Gao et al., 2021). We determined the levels of HYP and MMP-1 in the skin of control and UVB-treated mice. We found that the level of HYP was decreased and the expression of *Mmp-1* was increased in UVB-treated mice (Figure 1C). In addition, the expression levels of *p21* and *p53*, two genes closely related to skin aging (Chen Q. et al., 2020), were markedly increased in the skin of UVB-treated mice (Figure 1D). These results indicated that the mouse skin aging model was successfully established.

**FIGURE 2:**
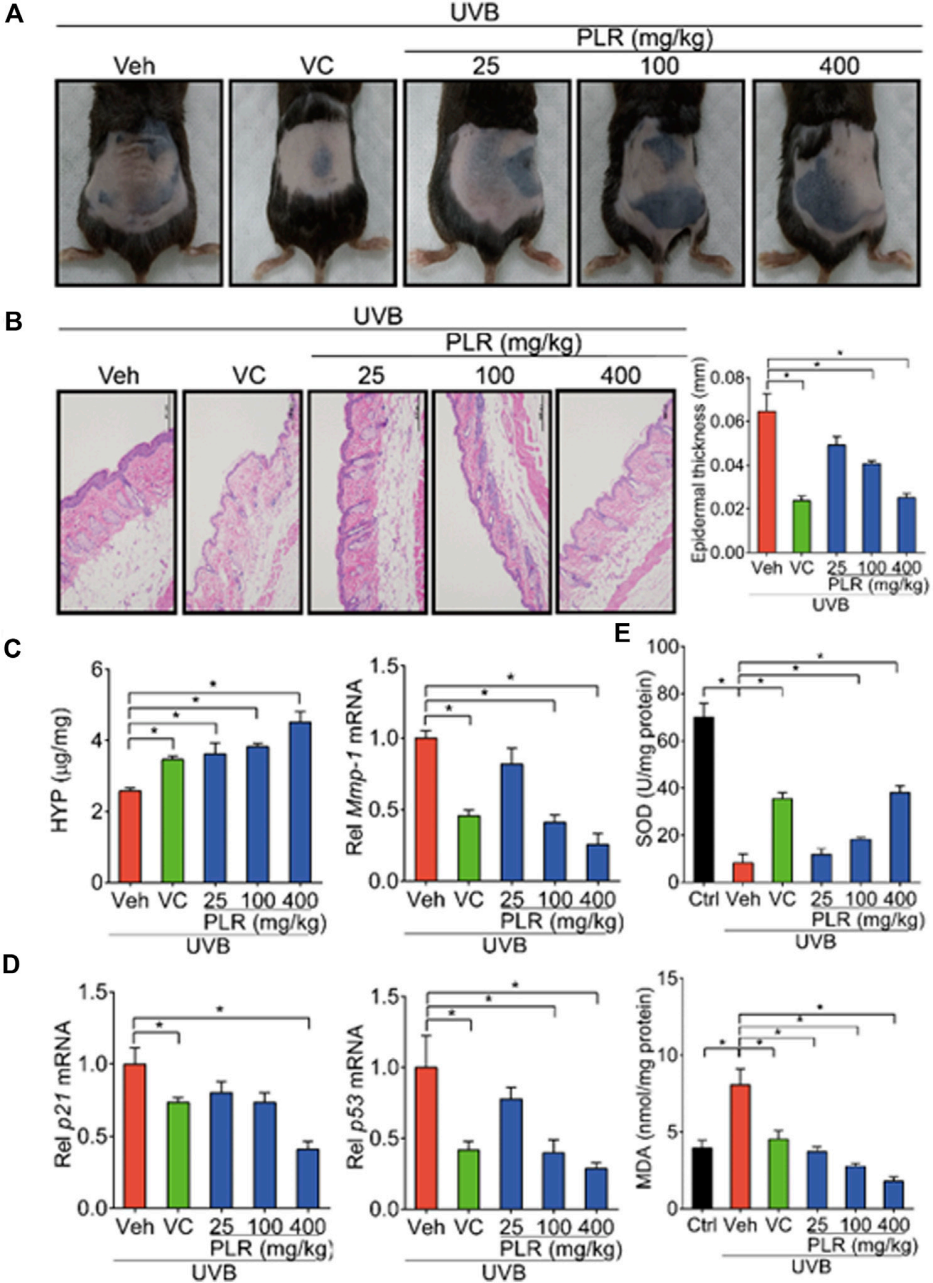
PLR shows dose-dependent protective effects on skin photoaging in mice. Mice were irradiated with UVB (120 mJ/cm^2^) for 45 days, with or without VC (40 mg/kg) or various doses of PLR extract (25, 100 or 400 mg/kg). **(A)** Photographs of mouse skin in the different groups. **(B)** Representative images of H&E staining and quantitative analysis of the epidermal thickness. Scale bar: 200 µm. **(C)** Content of HYP and the expression level of *Mmp-1* mRNA in the skin. **(D)** Expression levels of *p21* and *p53* mRNA in the skin. **(E)** Activity of SOD and level of MDA in the skin. Data are presented as mean ± SD (*n* = 6). *p < 0.05. Veh, vehicle.

Next, we tested the PLR efficacy against skin aging induced by UVB in mice. We found that skin wrinkles, epidermis thickness, and expression levels of *Mmp-1*/*p21*/*p53* were decreased, while the level of HYP was increased after treatment with PLR or VC (used as a positive control) in mice with UVB-induced skin aging (Figures 2A–D). Moreover, we determined whether SOD activity and MDA content (two indicators related to oxidation) were altered in mouse skin. We found that UVB inhibited SOD activity and increased the MDA level (Figure 2E). However, PLR dose-dependently promoted SOD activity and decreased the MDA level (Figure 2E). These results indicated dose-dependent protective effects of PLR on photoaging in mice.

### A protective effect of PLR on UVB-induced cell senescence

**FIGURE 3:**
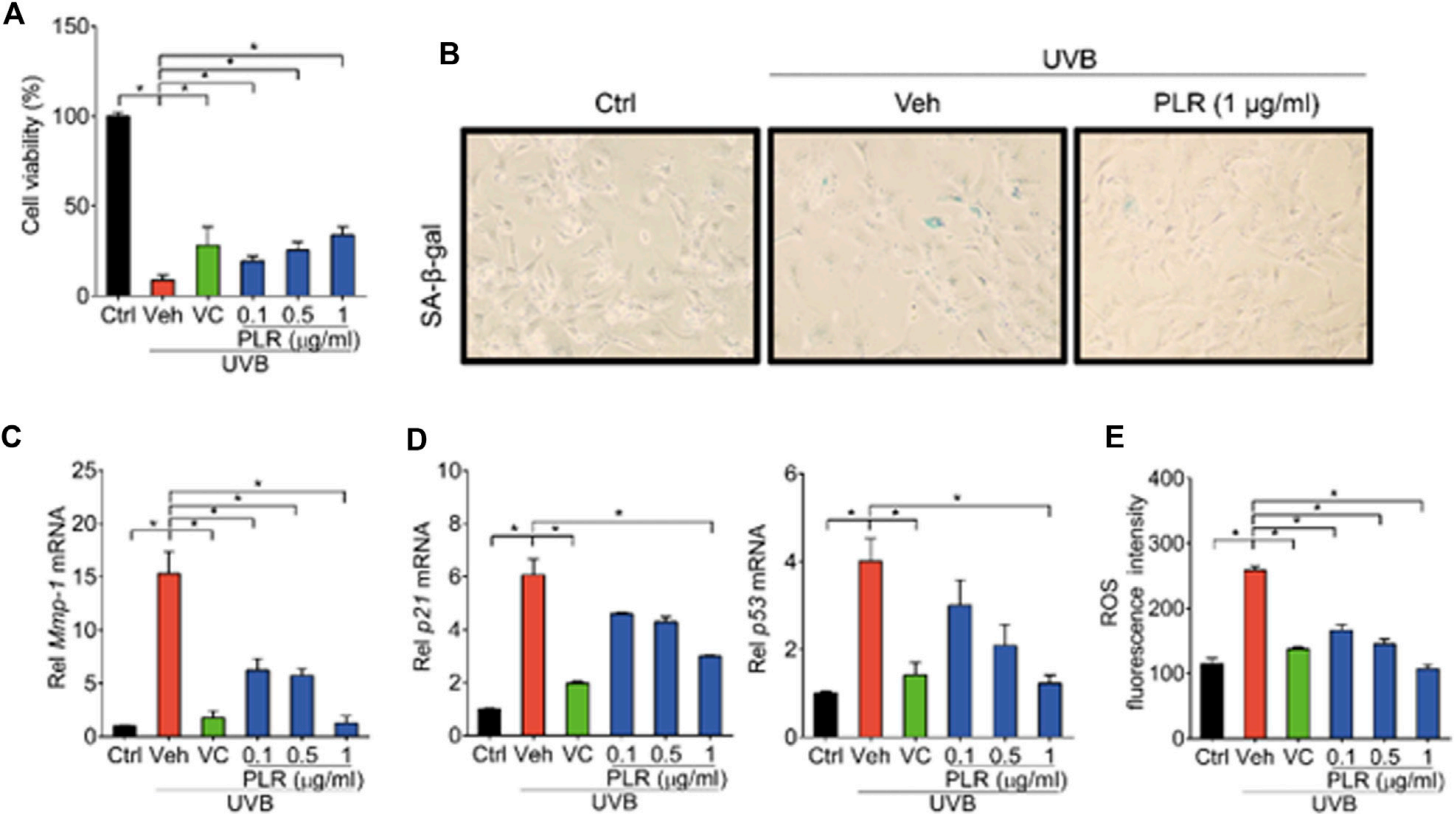
PLR shows dose-dependent protective effects on UVB-induced cell senescence. L929 cells were subjected to UVB irradiation (100 mJ/cm^2^) with or without VC (6 μg/ml) or various concentrations of PLR extract (0.1, 0.5 or 1 μg/ml). **(A)** Effects of PLR treatment on the viability of L929 cells. **(B)** Representative images of SA-β-gal staining. **(C,D)** Expression levels of *Mmp-1/p21/p53* mRNA in L929 cells. **(E)** ROS levels measured by DCFH-DA staining. Data are presented as mean ± SD (*n* = 3). *p < 0.05. Ctrl, control; Veh, vehicle.

To test whether PLR can relieve UVB-induced cell senescence *in vitro*, L929 cells were treated with VC (positive control) or various concentrations of PLR (0.1, 0.5 and 1 μg/ml) after exposure to UVB. In line with our expectations, UVB decreased cell viability, whereas PLR (in a dose-dependent manner) and VC significantly alleviated the inhibitory effect of UVB on cell viability (Figure 3A). In addition, PLR attenuated the induction of β-galactosidase activity by UVB (Figure 3B). RT-qPCR assay was applied to assess the regulatory effects of PLR on cell senescence-associated genes. Consistent with previous *in vivo* studies, the expression of *Mmp-1*/*p21*/*p53* was remarkedly induced after cell exposure to UVB, however, PLR (in a concentration-dependent manner) and VC significantly attenuated the induction effects of UVB on the expression of these genes (Figures 3C,D). Furthermore, we found that PLR and VC suppressed the induction of ROS in UVB-treated cells (Figure 3E). These data clearly indicated that PLR can inhibit UVB-induced cell senescence.

### PLR increases the expression of BMAL1 in aged skin

**FIGURE 4:**
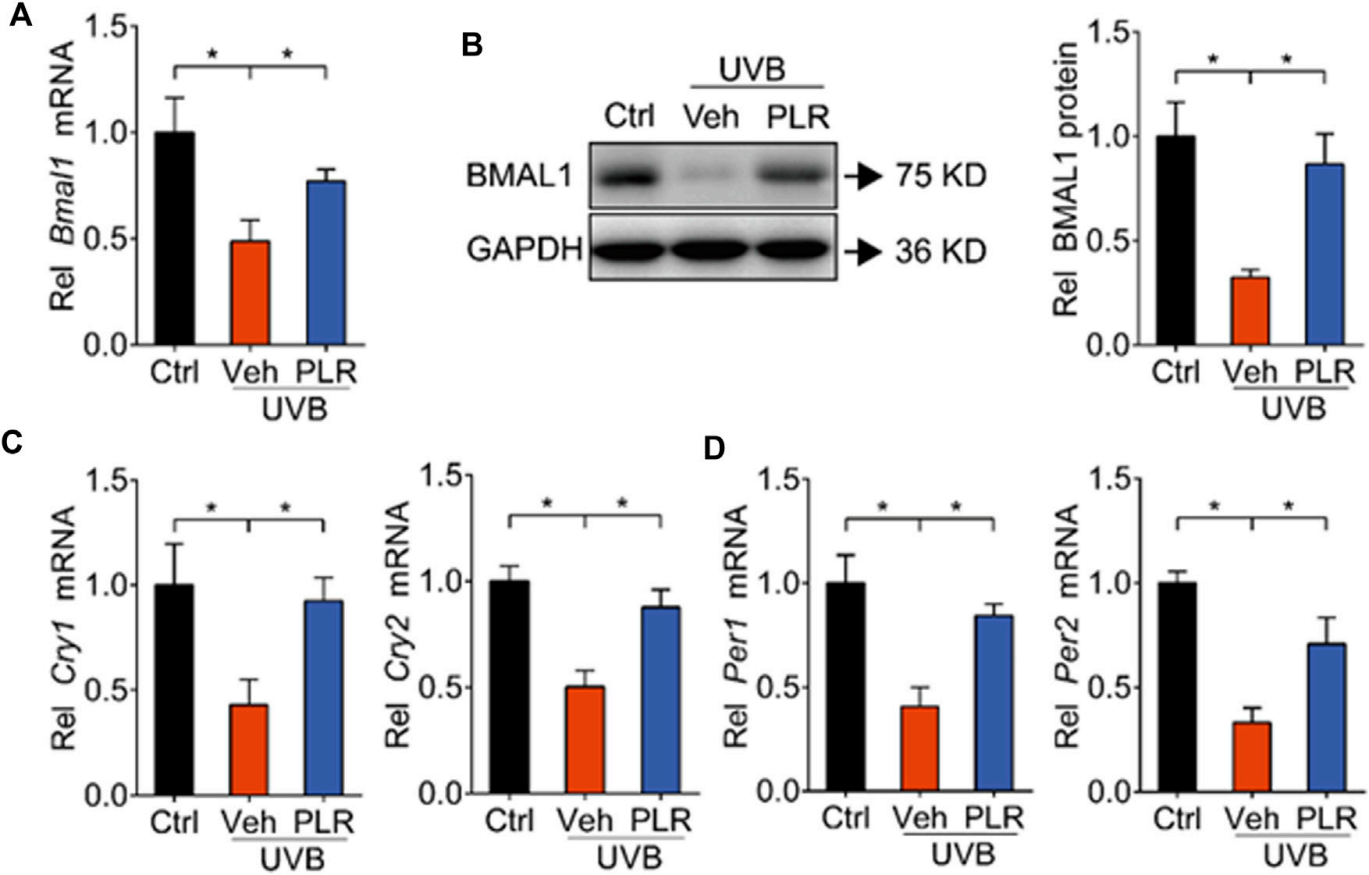
PLR modulates the expression of clock genes in aged skin. 400 mg/kg PLR was used to treat the skin of mice. RT-qPCR and Western blotting were carried out to determine the expression levels of core clock genes in mouse skin. **(A)** mRNA level of *Bmal1*. **(B)** protein level of BMAL1. **(C)** mRNA levels of *Cry1* and *Cry2*. **(D)** mRNA levels of *Per1* and *Per2*. Data are presented as mean ± SD (*n* = 6). *p < 0.05. Ctrl, control; Veh, vehicle.

**FIGURE 5:**
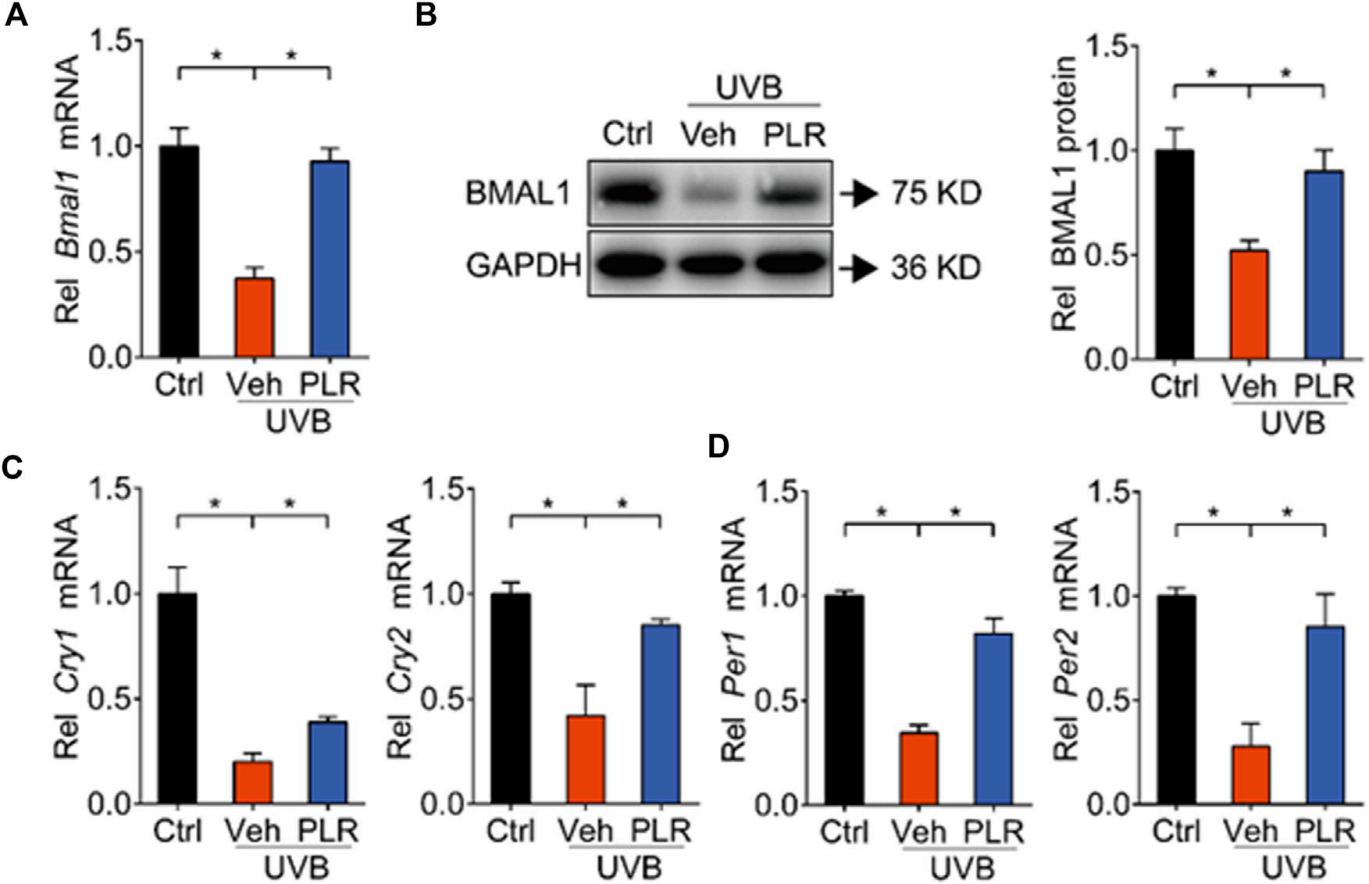
PLR modulates the expression of clock genes in senescent cells. 1 μg/ml PLR was used to treat the L929 cells. RT-qPCR and Western blotting were carried out to detect the expression levels of core clock genes. **(A)** mRNA level of *Bmal1*. **(B)** protein level of BMAL1. **(C)** mRNA levels of *Cry1* and *Cry2*. **(D)** mRNA levels of *Per1* and *Per2*. Data are presented as mean ± SD (*n* = 3). *p < 0.05. Ctrl, control; Veh, vehicle.

Because previous studies have identified BMAL1 as a key regulator of skin aging (Kondratov et al., 2006), we wondered whether PLR can modulate the expression of BMAL1 to alleviate UVB-induced skin aging. Compared with control mice, BMAL1 mRNA and protein levels were markedly decreased in mouse skin exposed to UVB (Figures 4A,B). In line with this, the expression levels of its target genes such as *Cry1*, *Cry2, Per1*, and *Per2* were decreased by UVB (Figures 4C,D). Interestingly, PLR treatment increased the expression of BMAL1 and its target genes in aged skin, thereby attenuating the inhibitory effects of UVB on BMAL1 (Figure 4). Likewise, PLR enhanced the expression of BMAL1 and its targets (*Cry1*, *Cry2, Per1* and *Per2*) in UVB-treated L929 cells (Figure 5). Altogether, these data indicated that PLR can up-regulate the expression of *Bmal1*, potentially contributing to its protective effect on UVB-induced aging.

### PLR increases expression of Nrf2 and antioxidant enzymes

**FIGURE 6:**
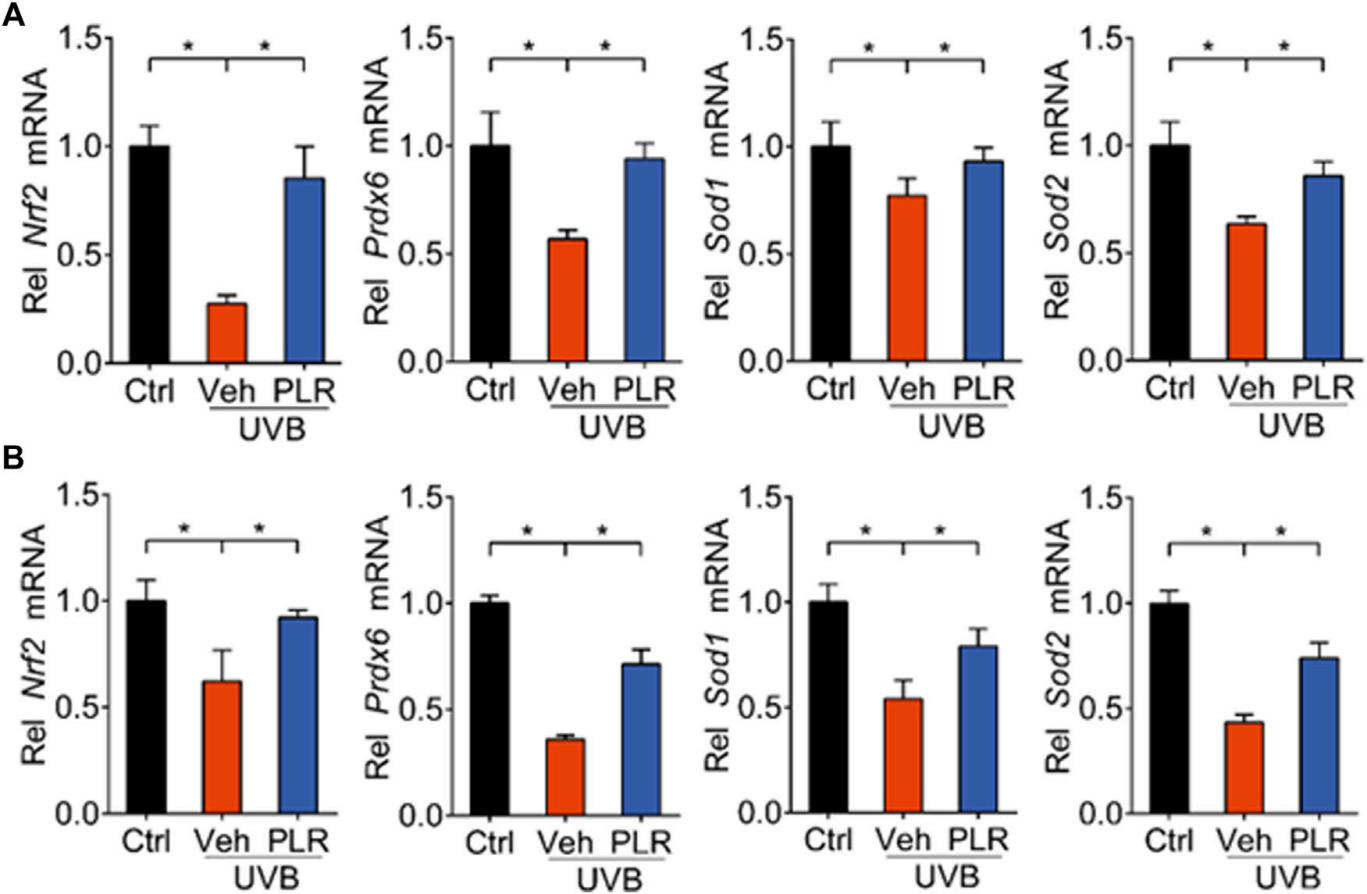
PLR increases expression of *Nrf2* and antioxidant genes. **(A)** Expression levels of *Nrf2*, *Prdx6*, *Sod1*, and *Sod2* mRNAs in mouse skin. 400 mg/kg PLR was used to treat the skin of mice. **(B)** Expression levels of *Nrf2*, *Prdx6*, *Sod1*, and *Sod2* mRNAs in L929 cells. 1 μg/ml PLR was used to treat the L929 cells. Data are presented as mean ± SD (*n* = 3). *p < 0.05. Veh, vehicle.

BMAL1 regulation of aging is attained mainly through modulation of oxidative stress and ROS homeostasis (Töbelmann and Dittmar, 2021; Zhang et al., 2022). BMAL1 promotes oxidative defense by up-regulating the expression of NRF2 (a major regulator of antioxidant enzymes) and of the major antioxidant enzymes (Early et al., 2018; Chhunchha et al., 2020). Given that PLR increases *Bmal1* expression, we next tested whether PLR also up-regulates the expression of NRF2 and major antioxidant enzymes. We found that the expression levels of *Nrf2*, *Prdx6, Sod1,* and *Sod2* were decreased in the skin of UVB-treated mice (Figure 6A). However, PLR treatment rescued the inhibitory effects of UVB on *Nrf2* and the antioxidant genes in mice with skin aging (Figure 6A). Likewise, PLR treatment led to increased mRNA levels of *Nrf2*, *Prdx6*, *Sod1*, and *Sod2* in UVB-treated L929 cells (Figure 6B). These results demonstrated that PLR increased the expression of *Nrf2* and antioxidant enzymes, consistent with its promoting effect on *Bmal1* expression.

### Identification of PLR as an antagonist of REV-ERBα

**FIGURE 7:**
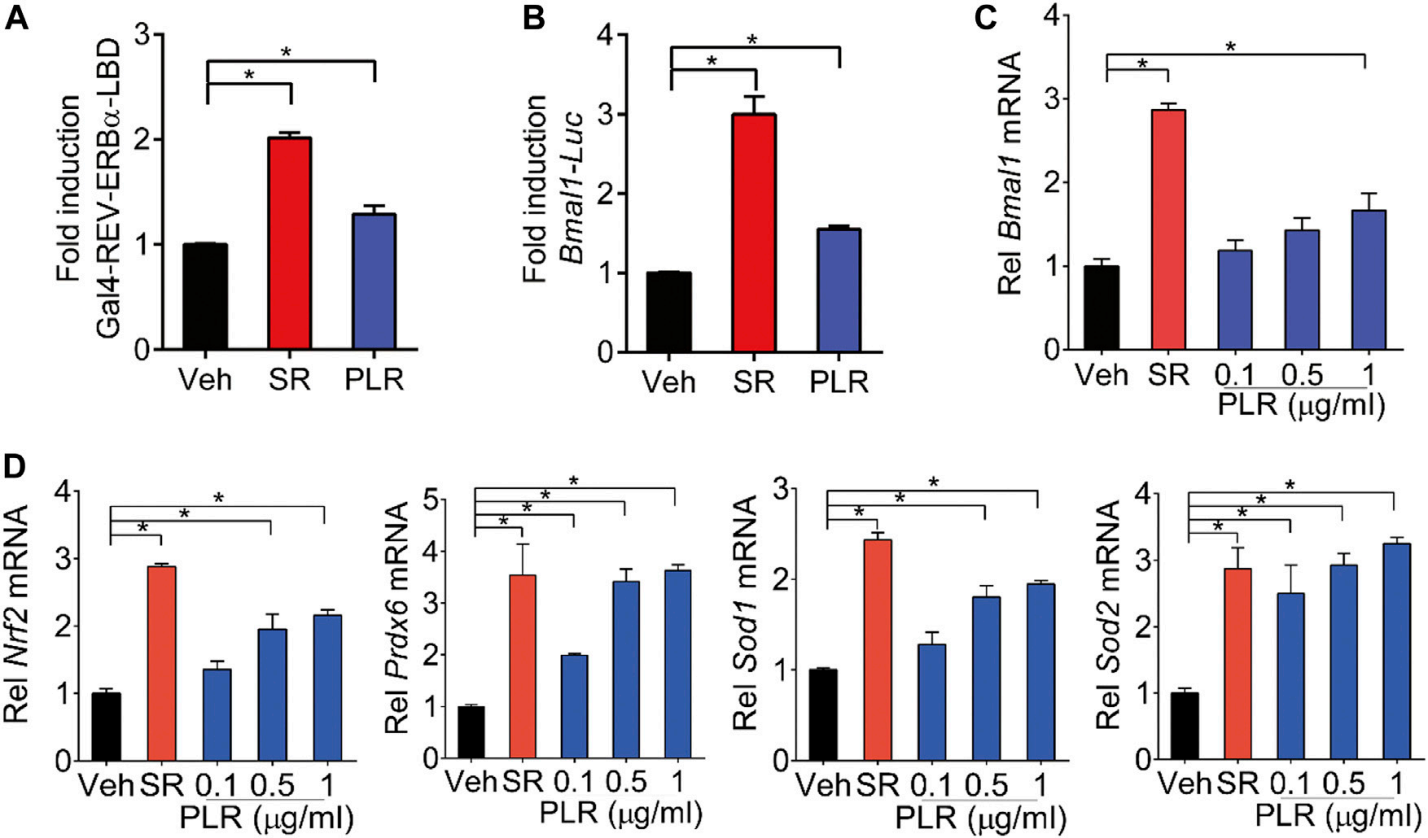
Identification of PLR as an antagonist of REV-ERBα. **(A)** SR8278 and PLR increase cellular luciferase activities in the Gal4-REV-ERBα-LBD chimeric assays. **(B)** Effects of PLR on *Bmal1-Luc* reporter activity in L929 cells. **(C,D)** mRNA levels of *Bmal1* and its target genes in L929 cells. Cells were treated with SR8278 (10 μM) or different concentrations of PLR as indicated for 24 h. Data are presented as mean ± SD (*n* = 3). *p < 0.05. Veh, vehicle; SR, SR8278.

REV-ERBα is a known negative regulator of BMAL1 and a druggable target (Burris, 2008; Patke et al., 2020). We wondered whether PLR regulates the expression of *Bmal1* through REV-ERBα. Like SR8278 (a known REV-ERBα antagonist), PLR increased the luciferase activities in the Gal4-REV-ERBα-LBD chimeric assays, indicating PLR as an antagonist of REV-ERBα (Figure 7A). Furthermore, PLR enhanced the *Bmal1* promoter activity according to a luciferase reporter assay in L929 cells (Figure 7B). Moreover, we found that PLR dose-dependently increased the expression of *Bmal1* and the genes involved in antioxidant responses such as *Nrf2*, *Prdx6*, *Sod1*, and *Sod2* (Figures 7C,D). Notably, PLR did not alter the viability and total RNA level of normal L929 cells (Supplementary Figure S2). Therefore, PLR was identified as a REV-ERBα antagonist which can induce *Bmal1* expression and promote antioxidant genes.

### REV-ERBα is required for the protective effect of PLR on skin aging

**FIGURE 8:**
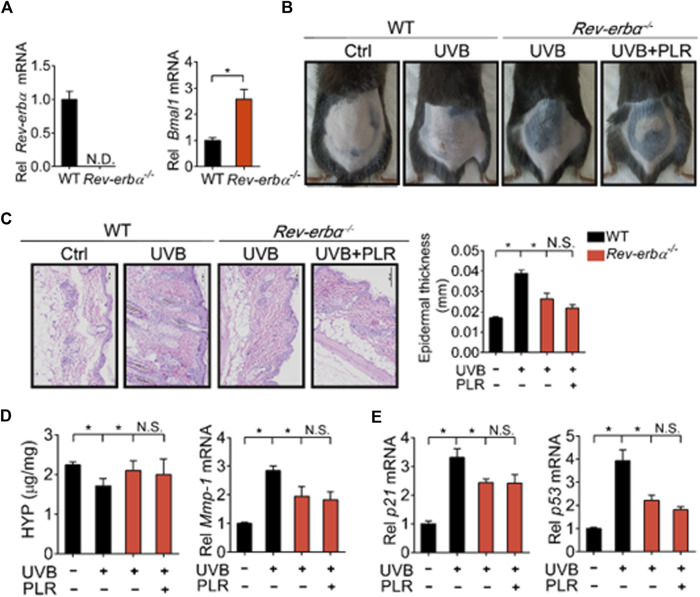
REV-ERBα is required for the protective effect of PLR on skin aging. **(A)** Knockout efficiency of *Rev-erbα*. **(B)** Photographs of mouse skin in the different groups. **(C)** Representative images of H&E staining and quantitative analysis of the epidermal thickness. Scale bar: 100 µm. **(D)** HYP content and *Mmp-1* mRNA level in mouse skin. **(E)** mRNA levels of *p21* and *p53* in the skin. 400 mg/kg PLR was used to treat the skin of mice. Data are presented as mean ± SD (*n* = 4). *p < 0.05. WT, wide type; N.D, not detected; N.S, not significant.

**FIGURE 9:**
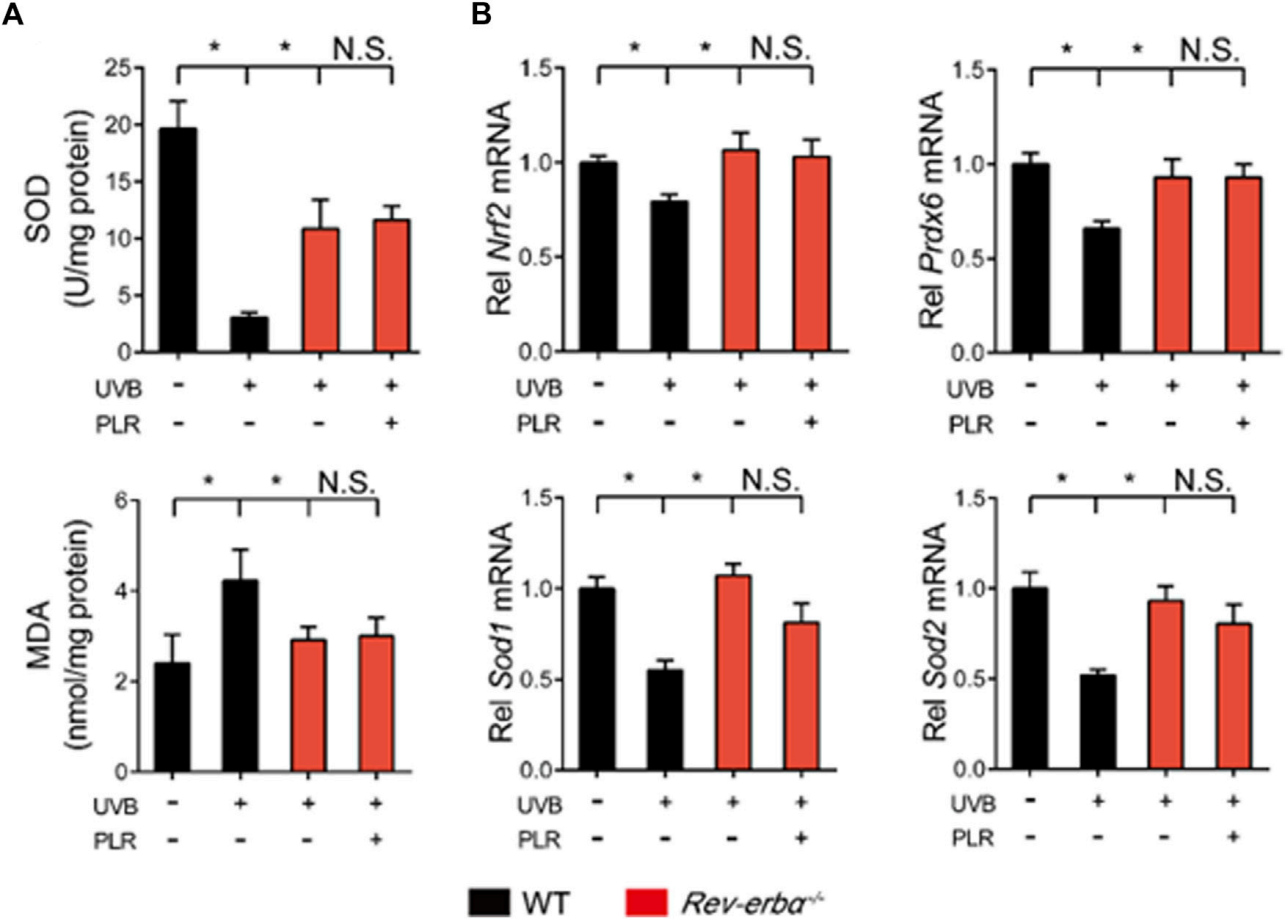
REV-ERBα is required for the protective effect of PLR on skin aging. **(A)** SOD activity and MDA level in the skin. **(B)** Expression levels of *Nrf2*, *Prdx6*, *Sod1*, and *Sod2* mRNA in the skin. 400 mg/kg PLR was used to treat the skin of mice. Data are presented as mean ± SD (*n* = 4). *p < 0.05. WT, wide type; N.S, not significant.

Lastly, we tested whether REV-ERBα is indeed necessary for the pharmacological effects of PLR on skin aging. We generated *Rev-erbα*-deficient (*Rev-erbα* ^−/−^) mice, and tested the PLR effect using this mouse line. As expected, *Rev-erbα* mRNA was absent and *Bmal1* was significantly increased in the skin of the knockouts (Figure 8A). *Rev-erbα* deficiency attenuated the UVB-induced skin aging phenotype due to enhanced *Bmal1* expression, as evidenced by reduced skin wrinkles and epidermal thickness as well as decreased *Mmp-1*, *p21* and *p53* expression but an increased HYP level (Figures 8B–E). In the meantime, *Rev-erbα* deficiency increased SOD activity and reduced the MDA level (Figure 9A) and caused elevations in mRNA levels of *Nrf2*, *Prdx6*, *Sod1*, and *Sod2* in mouse skin after UVB treatment (Figure 9B). In contrast, these skin properties did not change in *Rev-erbα*-deficient mice under a non-irradiated condition (Supplementary Figure S3). Remarkably, PLR showed no effect on UVB-induced skin aging in *Rev-erbα* ^−/−^ mice based on histological examinations and measurements of aging- and ROS-related markers as well as antioxidant factors (Figures 8, 9). Collectively, these results indicated that REV-ERBα as a drug target was required for the protective effect of PLR on UVB-induced skin aging.

## Discussion

We have identified PLR as a potential therapeutic agent for skin aging. PLR is a phytomedicine with little safety concern, and thus it is more acceptable in practical applications as compared with synthetic agents (e.g., retinoid). Moreover, we have observed that PLR treatment (topical application) of mice with UVB-induced photoaging led to increased skin expression of BMAL1 (an aging suppressor) and major antioxidant enzymes, as well as a decreased ROS level. The literature has established that ROS plays a critical role in skin damage and aging caused by UV radiation (Papaccio et al., 2022). UV-generated ROS leads to oxidative stress in the skin and thus to various cellular changes such as an increase in MMPs expression, enhanced collagen degradation and impaired collagen synthesis, which reflect major aspects of skin aging (Kim et al., 2018). Prior studies also revealed that BMAL1 protects from premature aging by up-regulating the expression of major antioxidant enzymes and promoting the antioxidant defense against ROS (Hardeland et al., 2003; Chhunchha et al., 2020). Treatment of *Bmal1* ^*−/−*^ mice with the antioxidant N-acetyl-l-cysteine rescues most prototypes of premature aging (Kondratov et al., 2009). In addition, PLR was identified as a REV-ERBα antagonist that can stimulate *Bmal1* transcription and expression (Figure 7). Loss of *Rev-erbα* in mice abrogated the protective effect of PLR on UVB-induced skin aging (Figures 8, 9). Therefore, we proposed that PLR ameliorates UVB-induced skin aging in mice by increasing skin expression of BMAL1 *via* antagonism of REV-ERBα and promoting the antioxidant defense.

There is accumulating evidence that circadian clock exerts a regulatory role in the aging process. Disrupted expression of clock genes can lead to early aging in animals. For instance, similar to *Bmal1* ^*−/−*^ mice, *Clock* ^*−/−*^ mice have a reduced lifespan and develop age-specific pathologies such as cataracts and dermatitis, suggesting an important role of CLOCK in aging (Dubrovsky et al., 2010). In contrast to the protective effects of BMAL1 and CLOCK on premature aging, PER2 (another clock component) is shown to promote premature aging (Levine et al., 2020). The mechanism of PER2 action involves the inhibition of BMAL1/CLOCK transactivation of *Sirt1* (sirtuin 1), a factor promoting longevity (or delayed aging) (Wang et al., 2016). On the other hand, aging is associated with disturbances in circadian rhythms such as altered sleep-wake cycle, hormonal arrhythmicity, and disrupted clock gene expression (Mattis and Sehgal, 2016). The bidirectional regulation between circadian clock and aging highlights the clock proteins as promising drug targets for aging, which is supported by current study in which PLR targets clock genes in mouse skin to ameliorate photoaging.

Because among clock components, BMAL1 (and its partner CLOCK) has a direct effect on skin aging (*via* direct regulation of antioxidant enzymes and thus of ROS elimination), it most likely acts to connect this skin disorder to circadian clock. Supporting this, *Bmal1* was markedly down-regulated in photoaged mouse skin (Figure 4). However, compared with the clock-aging linker BMAL1 and its partner CLOCK, REV-ERBα is a druggable target containing a ligand-binding domain accessible by small molecules. PLR is identified as a novel REV-ERBα antagonist (Figure 7). However, it is unknown whether PLR has an effect on REV-ERBβ, a paralog of REV-ERBα, which shares similar functions with REV-ERBα (Burris, 2008). Also, it remains unresolved which ingredients in PLR are responsible for the antagonistic effect. Nevertheless, it is speculated that the active constituent puerarin may be a major contributor as it can antagonize REV-ERBα to stimulate gene expression of its downstream targets (Chen M. et al., 2020).

The PLR efficacy against photoaging can be attributed to enhanced antioxidant defense and reduced ROS level due to up-regulation of BMAL1. However, there is a high possibility that ROS-independent mechanisms such as altered mTORC1 (mammalian target of rapamycin complex 1) signaling play an additional role in the anti-aging effect of PLR (Weichhart, 2018). This is because BMAL1 is shown to be a negative regulator of mTORC1 signaling, which promotes accelerated aging by regulating cell growth and proliferation (Khapre et al., 2014). In addition, we cannot exclude the possibility that BMAL1-independent pathways may contribute to the antioxidant effects of PLR. This is because PLR contains multiple types of antioxidants such as flavonoids that can directly act as scavengers of ROS and other free radicals (Bebrevska et al., 2010; Jin et al., 2012).

In this study, the PLR efficacy against skin aging was reflected by analyzing SOD activity, MDA/HYP levels, and *Mmp-1/p21/p53* expression. SOD acts as a major scavenger of superoxide anions in the body (Töbelmann and Dittmar, 2021). MDA is an indicator of lipid peroxidation (Tsikas, 2017). Therefore, SOD and MDA are thought to be two useful indicators of aged organism (Li et al., 2022). HYP is the most abundant amino acid in collagen, and its level indirectly reflects the total collagen content (Wu et al., 2019). MMP-1 is a collagenase that plays a key role in degradation of dermal collagen during skin aging (Kim et al., 2018). Thus, HYP and MMP-1 can be used as markers of skin aging process. *p21* and *p53* are anti-oncogenes that promote cell senescence (Chen Q. et al., 2020), and are also regarded as markers of skin aging. In fact, measurements of SOD activity, MDA/HYP levels, and *Mmp-1/p21/p53* expression have been widely performed to assess aging and to screen anti-aging agents (Sharpless, 2015; Lee et al., 2016; Sun et al., 2018; Li et al., 2022; Liu et al., 2022).

## Conclusion

The herbal medicine PLR has a protective effect on skin aging induced by UVB in mice. Mechanistically, PLR antagonizes REV-ERBα and increases skin BMAL1 (an aging-inhibiting factor) expression to attenuate photoaging.

## Data Availability

The original contributions presented in the study are included in the article/Supplementary Material, further inquiries can be directed to the corresponding authors.

## Abbreviations

AP-1, activation protein 1; BMAL1, brain and muscle ARNT-like protein 1; CLOCK, circadian locomotor output cycles kaput; CRY, cryptochrome; GPX, glutathione peroxidase; HYP, hydroxyproline; MDA, malondialdehyde; MMPs, matrix metalloproteinases; mTORC1, mammalian target of rapamycin complex 1; NRF2, nuclear factor erythroid 2-related factor 2; PER1/2, period 1/2; PLR, Puerariae lobatae radix; PRDXs, peroxiredoxines; ROS, reactive oxygen species; RT-qPCR, reverse transcription-quantitative polymerase chain reaction; SA-β-gal, senescence-associated-β-galactosidase; SIRT1, sirtuin 1; SOD, superoxide dismutase; UVB, ultraviolet-B; VC, vitamin C.
