# Supplementary material for: Puerariae lobatae radix protects against UVB-induced skin aging via antagonism of REV-ERBα in mice
Source: Front Pharmacol. 2022 Dec 22;13:1088294. doi: 10.3389/fphar.2022.1088294 (PMC9813444; doi:10.3389/fphar.2022.1088294)
Supplement: Supplementary file 1 [file DataSheet2.PDF]

Supplementary material

LC-MS/MS analysis

The main constituents of PLR extract were analysed and quantified by a LCMS-8045 mass spectrometer (Shimadzu, Kyoto, Japan) equipped with an electrospray ionization source. High-performance liquid chromatographic separation was performed with a Nexera XP UHPLC system (Shimadzu, Kyoto, Japan) coupled with a Shim-pack GIST C18 column (100 mm×2.1 mm, 2.1 μm). The sample injection volume was 2 μL. The mobile phases consisted of water (A) and acetonitrile (B) acidified with 0.1% formic acid. Flow rate was set at 0.25 ml/min. Gradient elution program was 20-90% B (0-3 min), 90% B (3-4 min), 90-20% B (4-4.5 min), and 20% B (4.5-6 min). The working conditions of LCMS-8045 system were as follows: ESI interface temperature at 300 °C, heat block temperature at 400 °C, DL temperature at 250 °C, heating gas flow at 10 L/min, nebulizing gas flow at 3 L/min, and drying gas flow at 10 L/min. To characterize the chemical fingerprint of PLR, mass spectrometer was operated at positive and negative ion scan modes. Puerarin and daidzein were quantified using the multiple reaction monitoring (MRM) mode with positive ion detection. The mass transition ion pairs and contents of puerarin and daidzein in PLR exact are provided in supplementary Table S1.

Table S1: LC-MS/MS determination of puerarin and daidzein in PLR extract.

Constituents	RT (min)	Transition ion pair	Molecular formula	Content (mg/g)
Puerarin	2.07	417.35→297.30	$C_{21}H_{20}O_9$	207.5
Daidzein	4.18	255.15→199.12	$C_{15}H_{10}O_4$	31.6

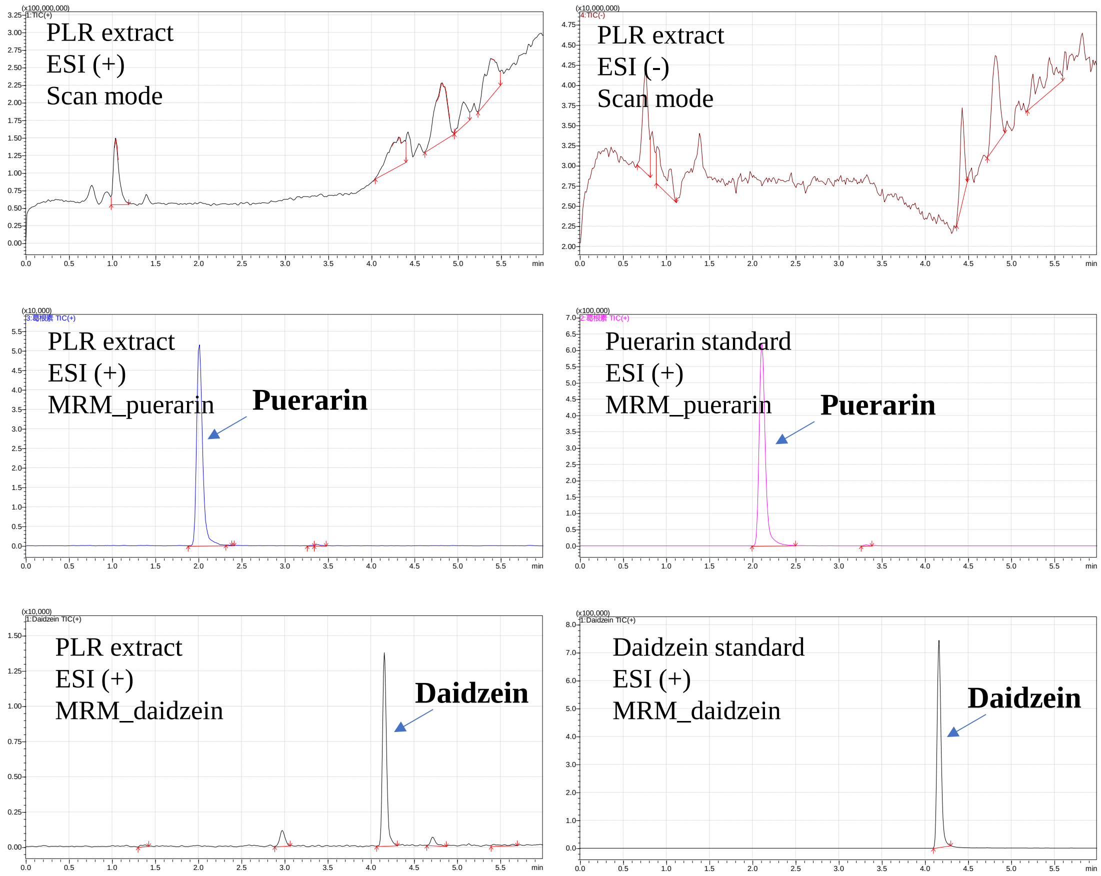

Figure S1. Representative LC-MS chromatograms for PLR extract.

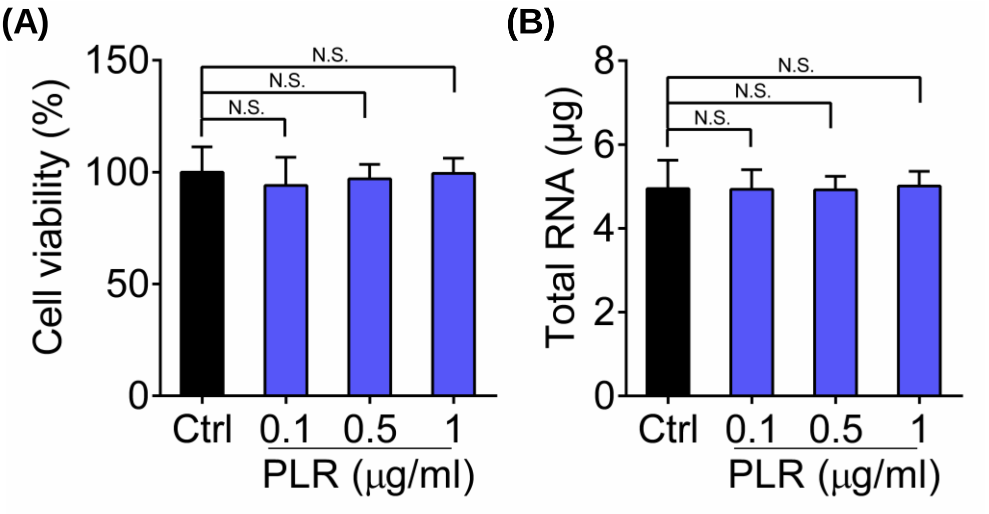

Figure S2. PLR does not alter the viability and total RNA level of normal L929 cells. (A) Effect of PLR on the viability of L929 cells. (B) Effect of PLR on total RNA level of L929 cells. L929 cells were treated with or without PLR extract (0.1, 0.5 or 1 μg/mL) for 24 h. Data are presented as mean ± SD (n = 3). *P < 0.05. Ctrl, control; N.S., not significant.

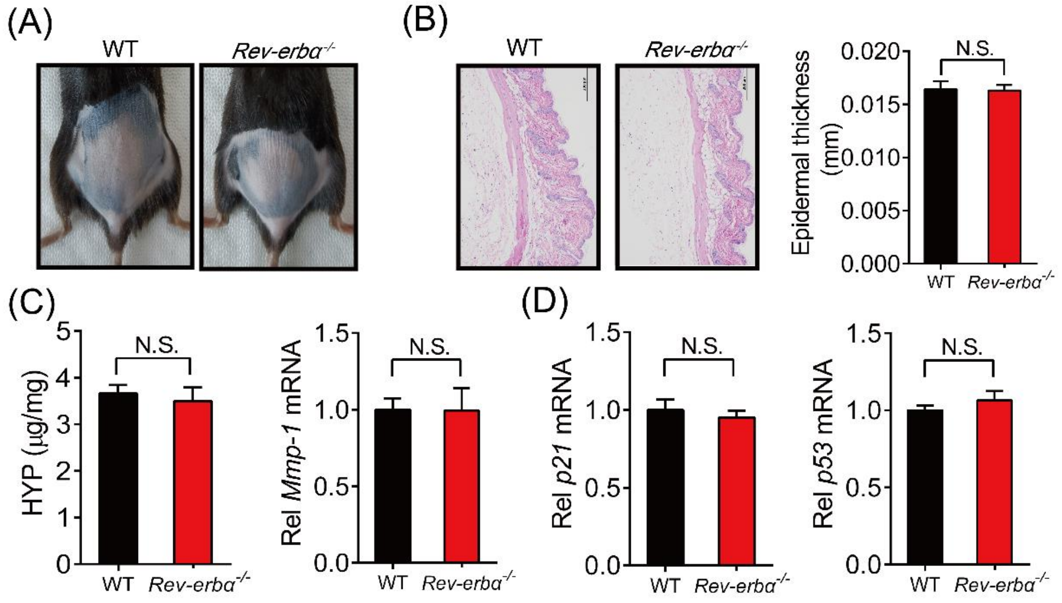

Figure S3. Comparisons of skin properties between *Rev-erbα*$^{-/-}$ and WT mice under a non-irradiated condition. (A) Photographs of the back skin of mice. **(B)** Representative images of H&E staining and quantitative analysis of the epidermal thickness. Scale bar: 100 μm. **(C)** Content of HYP and expression level of *Mmp-1* mRNA in the skin. **(D)** Expression levels of *p21* and *p53* mRNA in the skin. Data are presented as mean ± SD (n = 3). *P < 0.05. WT, wide type; N.S., not significant.
