# Supplementary figures and images for: Puerariae lobatae radix protects against UVB-induced skin aging via antagonism of REV-ERBα in mice
Source: Front Pharmacol. 2022 Dec 22;13:1088294. doi: 10.3389/fphar.2022.1088294 (PMC9813444; doi:10.3389/fphar.2022.1088294)

Figure 4B

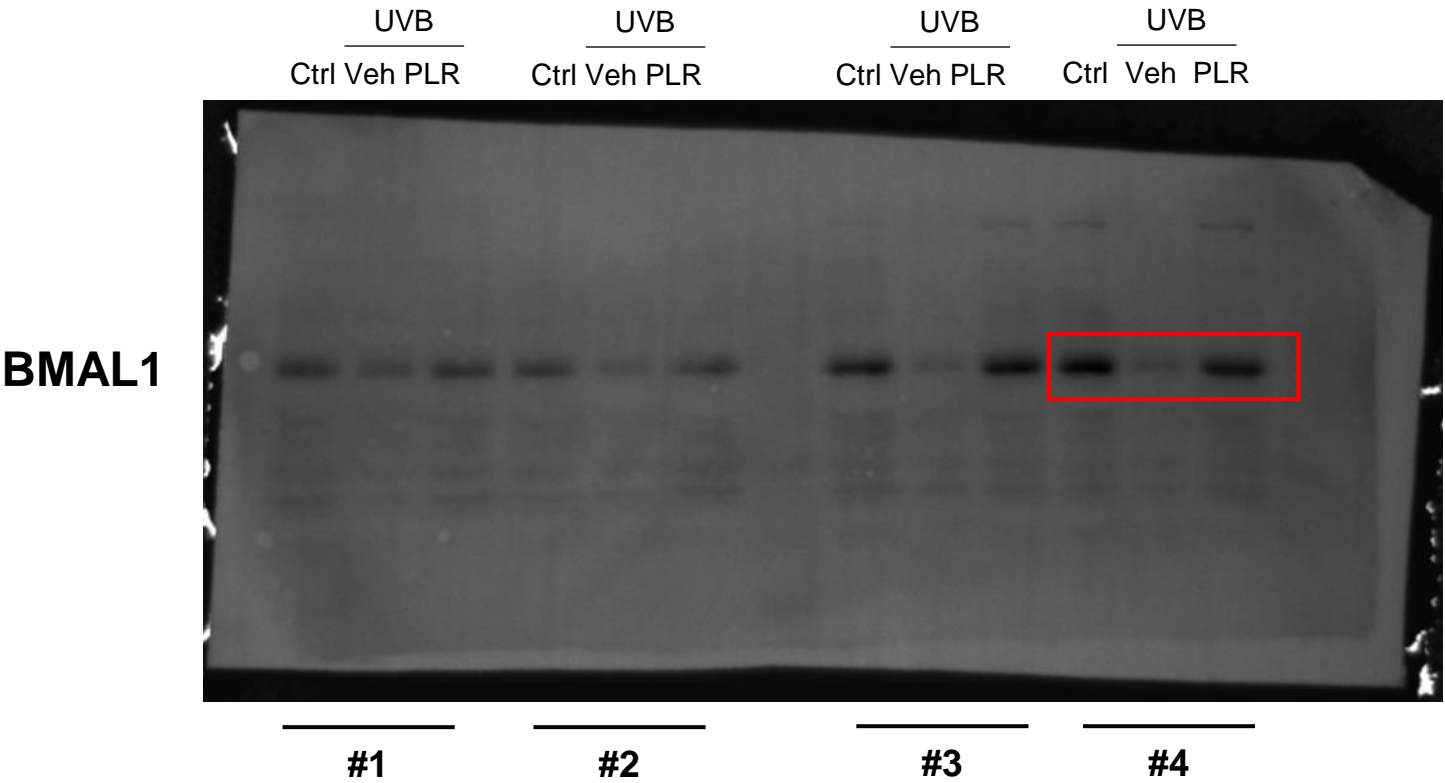

Figure 4B

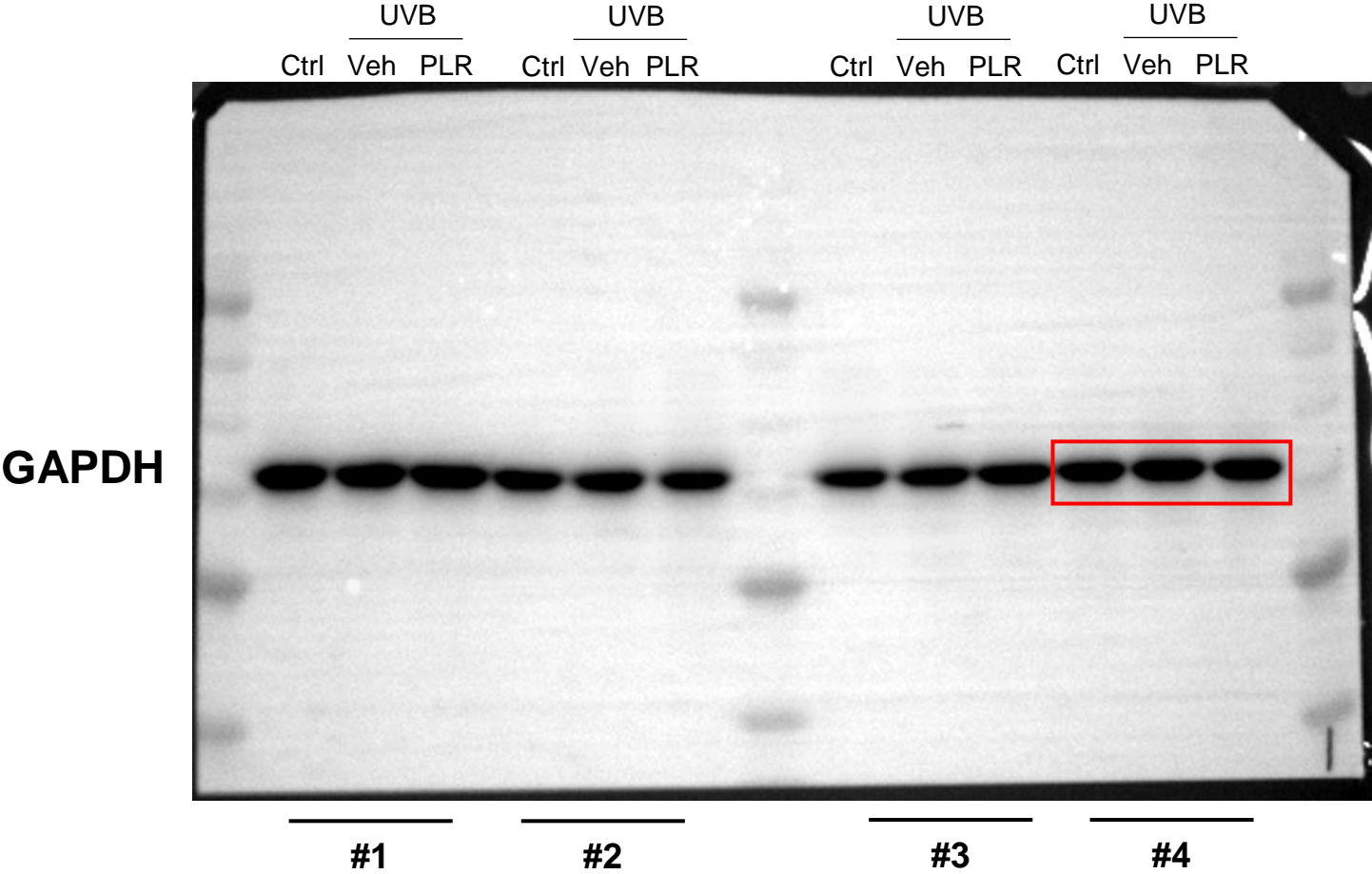

Figure 5B

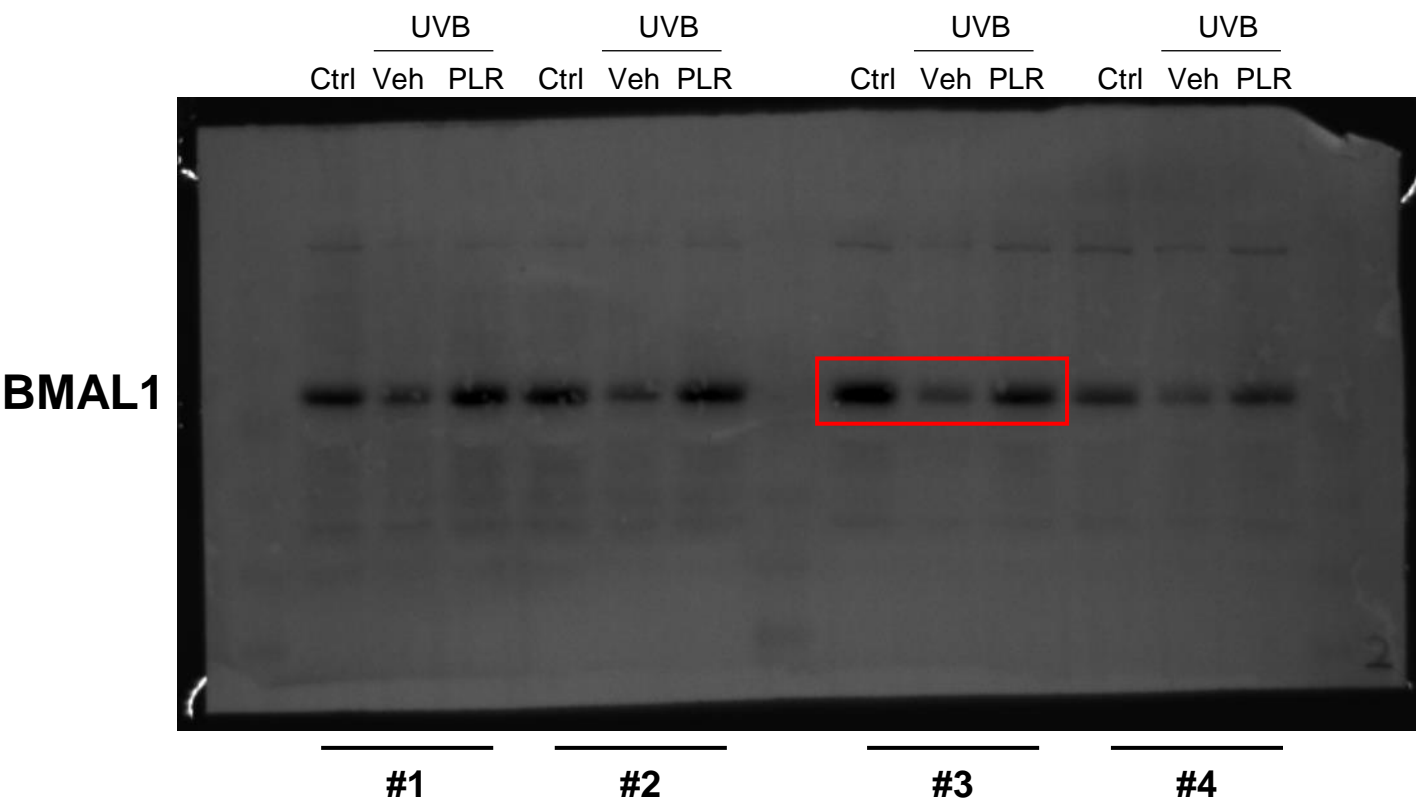

Figure 5B

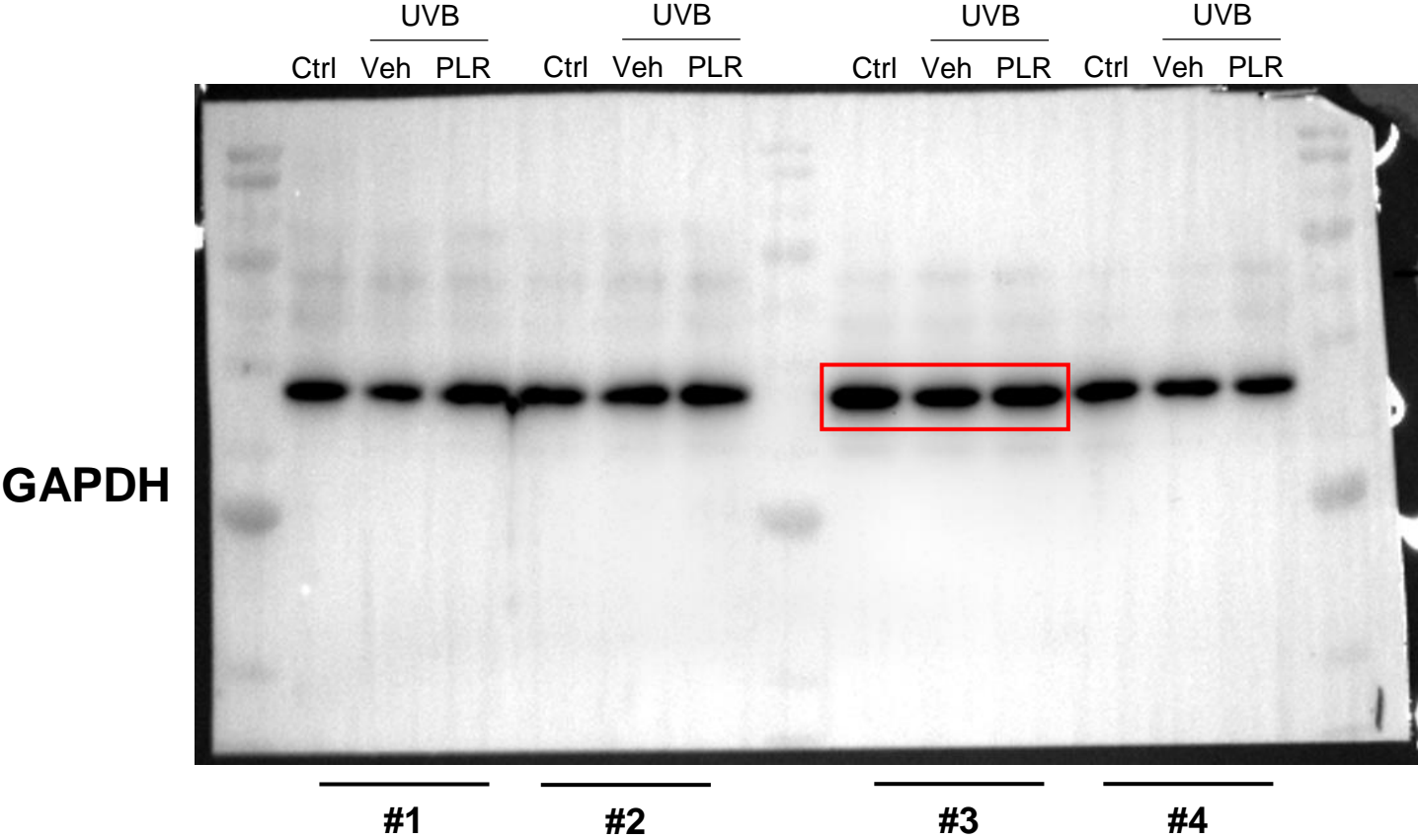

Supplement: Supplementary file 2 [file Image1.PDF]
